# Influence of recreational noise on temporal fine structure processing in college going students

**DOI:** 10.1016/j.bjorl.2026.101927

**Published:** 2026-10-17

**Authors:** Visishta Thavarayil, Leelavathi Thamizhmani, Kanaka Ganapathy, Hari Prakash Palaniswamy

**Affiliations:** a Faculty of Audiology and Speech Language Pathology, Sree Balaji Medical College and Hospital, Chennai, India; b Department of Speech and Hearing, Manipal College of Health Professions, Manipal Academy of Higher Education, Manipal, India

**Keywords:** Recreational noise, Temporal fine structure processing, Hidden hearing loss, Speech perception in noise, Amplitude modulation detection

## Abstract

**Objectives:**

The prevalence of noise exposure due to recreational activities and usage of Personal Listening Devices (PLD) is rising. This study investigated the effects of recreational noise exposure on temporal auditory processing in young adults with clinically normal hearing.

**Methods:**

A total of 123 college students aged 18- to 25-years, all of whom exhibited normal Pure Tone Audiometry (PTA) results, were enrolled in a cross-sectional study. Recreational noise exposure was estimated using the Exposed Noise and Hearing Disorders of Conscripts (ENHDC) questionnaire, and participants were categorised into low and high exposure groups. Auditory processing was assessed through three behavioural tasks: 1) Speech Perception in Noise (SPIN), using Kannada sentences presented in four-talker babble to determine Signal to Noise Ratio (SNR-50); 2) Temporal Fine Structure ‒ Adaptive Frequency (TFS-AF), to evaluate binaural phase sensitivity; and 3) Amplitude Modulation Detection (AMD), using a 5 kHz tone modulated at 19 Hz to evaluate temporal envelope processing.

**Results:**

Non-parametric analyses revealed significantly poorer SPIN scores in the high-exposure group (p = 0.035), alongside marginal but statistically significant elevations in PTA thresholds for both ears (p < 0.05), though still within clinical norms. No significant group differences were observed in TFS-AF (p = 0.737) or AMD (p = 0.153), although AMD thresholds were slightly elevated in the high exposure group. Extended High Frequency Audiometry (EHFA) findings showed a non-significant increase in elevated thresholds above 8 kHz.

**Conclusion:**

These results suggest that recreational noise exposure may produce subtle deficits in suprathreshold auditory processing before measurable hearing loss occurs. Early identification using speech-in-noise tasks and behavioural temporal assessments may support preventive strategies to safeguard auditory health in at-risk populations.

**Level of Evidence:**

Level 3.

## Highlights

• Hidden hearing loss detected in young adults with clinically normal audiograms.
• Speech-in-noise deficits linked to recreational noise exposure in college students.
• Personal listening devices contribute most to cumulative noise exposure patterns.
• SPIN testing more sensitive than audiometry for detecting early cochlear damage.

## Introduction

With over 1.1 billion young people worldwide at risk for hearing loss from unsafe listening practices, recreational noise exposure has emerged as a silent epidemic among today's youth.^1^ While prolonged or intense noise exposure has long been recognized as a well-established cause of sensorineural hearing loss in occupational settings such as factories, construction sites, and mining operations,^2^^,^^3^ increasing attention has turned toward non-occupational sources of noise, particularly those encountered during leisure activities. This shift has led to the identification of Recreational Noise-Induced Hearing Loss (RNIHL) as an emerging public health concern that demands urgent investigation.^4^, ^5^, ^6^

Recreational noise exposure is pervasive among adolescents and young adults. Common sources include concerts, nightclubs, music festivals, and personal audio systems such as smartphones and MP3 players. Recent global estimates highlight that unsafe listening practices from personal listening devices are highly prevalent among adolescents and young adults.^7^ These devices can produce sound levels exceeding 100 dB SPL, especially when used in noisy environments or at high volumes.^6^ Live events may expose listeners to sound levels greater than 110 dB SPL,^8^ and musical practice among students often lacks monitoring or hearing protection.^9^^,^^10^ Cultural norms, limited risk perception, and inconsistent use of hearing protection further exacerbate vulnerability in this population.^11^, ^12^, ^13^ Objective measurements of listening device sound levels have shown that many young users exceed recommended safe exposure limits during daily listening routines.^14^

Exposure to high-level sounds can result in Temporary Threshold Shifts (TTS), which may resolve with rest but can lead to Permanent Threshold Shifts (PTS) and irreversible damage with repeated exposure.^15^^,^^16^ There is increasing concern that cochlear damage can occur even when audiometric thresholds are within normal limits. This condition, termed cochlear synaptopathy, involves the loss of synaptic connections between inner hair cells and auditory nerve fibers^17^^,^^18^ and may manifest as Hidden Hearing Loss (HHL). HHL is characterized by suprathreshold auditory deficits despite normal pure-tone thresholds. It has been linked to selective damage of low Spontaneous Rate (SR) auditory nerve fibers, which are critical for processing complex acoustic signals such as amplitude modulation and Temporal Fine Structure (TFS).^19^ As a result, individuals with HHL may experience difficulty understanding speech in noise or tracking rapid changes in sound despite clinically normal audiograms. Recent review emphasize ongoing uncertainty regarding the clinical detectability of hidden hearing loss in humans, despite strong mechanistic evidence.^20^

However, a critical gap exists in our understanding of how recreational noise exposure affects young listeners. While traditional audiometry may show normal thresholds, subtle auditory processing deficits may already be present, potentially serving as early indicators of cochlear damage. Young adults may be suffering from a gradual hearing loss that goes unnoticed by standard clinical evaluations, putting them at risk of continued exposure and further decline.^21^^,^^22^ Behavioral measures such as Speech Perception In Noise (SPIN), Temporal Fine Structure (TFS) sensitivity, and Amplitude Modulation Detection (AMD) have been proposed as more sensitive tools for detecting subclinical auditory deficits.^23^ Studies have shown that individuals with high recreational noise exposure perform poorly on SPIN and TFS tasks^21^^,^^24^ though findings across studies remain inconsistent due to variability in exposure quantification and individual susceptibility.^25^^,^^26^

Given the high prevalence of Personal Listening Device (PLD) usage and the early onset of symptoms such as tinnitus and auditory fatigue among college students, these findings underscore the importance of evaluating subtle auditory changes in this population.^27^ Therefore, the present study aims to explore the influence of recreational noise exposure on temporal fine structure processing and speech perception in noise among college students with clinically normal hearing. The study specifically investigates whether cumulative noise exposure is associated with subtle suprathreshold auditory deficits that may serve as early indicators of cochlear synaptopathy. Identifying these changes may support early intervention and help develop preventive strategies for preserving auditory health in high-risk populations.

## Methods

### Participants

A total of 123 college students (82 females and 41 males) aged 18–25 years were recruited from Manipal University using purposive sampling. Inclusion criteria required normal hearing thresholds (≤ 20 dB HL) across 0.25–20 kHz. A thorough case history was taken, and participants with a history of otologic disease, hearing loss, formal music training, occupational noise exposure, or loud noise exposure in the past 24 h were not included in the study. Individuals reporting temporary symptoms such as tinnitus, muffled hearing, or ear fullness were also excluded from testing.

### Ethics

The study was approved by the Institutional Ethics Committee (IEC) (IEC2:345/2024) of Kasturba Medical College (KMC) and Kasturba Hospital (KHC), Manipal, India.

### Procedure

All participants underwent otoscopy and Pure Tone Audiometry (PTA) using a Madsen Astera audiometer (Otometrics, Natus Medical Inc.) with RadioEar DD450 headphones. Air conduction thresholds were obtained at octave and inter-octave frequencies between 250 Hz and 8 kHz using a modified Hughson-Westlake procedure. Pure Tone Averages (PTA) were computed using thresholds at 500 Hz, 1 kHz, and 2 kHz, while High Frequency Averages (HFA) were calculated using thresholds at 2, 4, and 8 kHz. Extended high-frequency audiometry (EHFA) was also performed, ranging from 9 kHz to 20 kHz and averaged across all test frequencies. Followed by this, auditory processing abilities were assessed using Speech Perception in Noise (SPIN), Temporal Fine Structure – Adaptive Frequency (TFS-AF), and Amplitude Modulation Detection (AMD). Additionally, recreational noise exposure was assessed using the Exposed Noise and Hearing Disorders of Conscripts (ENHDC) questionnaire.

#### Exposed noise and hearing disorders of conscripts (ENHDC)

ENHDC questionnaire^28^ collected detailed information on 19 recreational noise activities, including PLD use, concerts, gaming, fireworks, and motor sports. For each activity, participants reported perceived loudness on a 5-point scale, frequency of engagement, average session duration, total years of exposure, and the use of hearing protection. These values were used to calculate the equivalent weekly exposure level (Leq,w) using the formula: Leq,w=LAeq+10log₁₀(Tw/To); where *LAeq* represents the loudness estimate in dBA, *Tw* is time spent weekly, and *To* is the reference duration (40-hs). Cumulative exposure (Leq,cum) was estimated as Leq,cum=LAeq,w+10log₁₀(Tyear).

### Auditory processing measures

1 Speech Perception in Noise (SPIN)

SPIN was assessed using sentence lists in Kannada, presented in a four-talker babble masker. Sentences were delivered binaurally via MATLAB R2023b through calibrated headphones. Participants were instructed to repeat each sentence aloud. An adaptive 2 dB step up-down staircase procedure was used, beginning at +10 dB Signal to Noise Ratio (SNR). The SNR was adjusted based on participant responses, with eight reversals per run. The SNR50, defined as the SNR at which 50% of the sentences were correctly identified, was calculated as the average of the final four reversals. This approach has been shown to reliably quantify speech-in-noise performance in young adults.^24^

2 Temporal Fine Structure – Adaptive Frequency (TFS-AF)

TFS sensitivity was measured using the TFS-AF method adapted from Bharadwaj et al. (2015).^21^ A two-Interval Forced-Choice (2IFC) paradigm was employed to estimate the upper frequency limit for detecting Interaural Phase Differences (IPDs). Each trial included two intervals: one with four diotic tones (no phase shift) and one with tones alternating between 0° and 180° phase shifts across ears. Stimuli were presented over headphones. The test was carried out using a custom script in MATLAB R2023b. Participants were instructed to indicate which interval contained the “moving” or spatially displaced percept. A 2-up/1-down adaptive staircase tracked the Highest frequency (Hz) at which phase shifts could be reliably detected.

### Amplitude modulation detection (AMD)

AMD was evaluated using a three-interval, Three-Alternative Forced-Choice (3AFC) paradigm in PsychoPy (Version: 2021.1.4), following the procedure described by Stone and Moore (2014).^29^ In each trial, three 5 kHz carrier tones were presented in narrowband noise centred at 5 kHz. One interval contained an Amplitude-Modulated (AM) tone at 19 Hz, while the remaining two intervals contained unmodulated tones. Participants were instructed to identify the interval that was perceptually different. Modulation depth was varied adaptively using the Parameter Estimation by Sequential Testing (PEST) algorithm. The threshold was defined as the average modulation depth (in dB) across the final six reversals.

### Statistical analysis

All data were analysed using Jamovi v2.3.28. Normality was assessed using the Shapiro–Wilk test. For examining the impact of cumulative noise exposure, participants were stratified into quartiles based on cumulative exposure scores calculated from the ENHDC questionnaire. Comparisons were made between the lowest (Q1) and highest (Q4) quartiles using the Mann-Whitney *U*-test. Spearman’s rank correlation was used to assess associations between cumulative noise exposure (Leq, cum) and auditory outcomes (SPIN, TFS-AF, AMD). *Z*-scores were used for correlational analyses when appropriate. Significance was set at p < 0.05. Effect sizes were reported where applicable using rank biserial correlation and epsilon squared.

## Results

A total of 123 college-going students participated in the study, including 82 females and 41 males. Participants ranged in age from 18- to 25-years (M = 21, SD = 2.06). The majority were undergraduates (n = 103). All the participants reported regular exposure to recreational noise, primarily using Personal Listening Devices (PLDs).

### Exposed noise and hearing disorders of conscripts (ENHDC) questionnaire

Participants reported involvement in a range of indoor and outdoor noise-generating activities. The most frequently reported sources of exposure included PLD use (89.4%), watching movies or attending the theatre (80.5%), and fireworks or impulse noise (74.8%). In contrast, exposure to traffic noise (11.4%) and construction environments (1.6%) was least common (Table 1).

**Table 1:** Self-reported participation in recreational noise-related activities among college students (n = 123).

Activity Type	Activity	n (%)
Indoor Activities	Watching television	81 (65.9%)
	Playing video games	29 (23.6%)
	Watching movies/theatre	99 (80.5%)
	Attending indoor sports events	24 (19.5%)
	Exercising to music	44 (35.8%)
	Using indoor tools	19 (15.4%)
Outdoor Activities	Playing in stage or orchestra	49 (39.8%)
	Musical instruments	21 (17.1%)
	Going to nightclubs or pubs	40 (32.5%)
	Attending concerts, festivals, or musical events	85 (69.1%)
	Fireworks or impulse noise exposure	92 (74.8%)
	Construction noise	2 (1.6%)
	Traffic noise	14 (11.4%)
PLD Usage	Listening via loudspeakers	50 (40.7%)
	Listening via headphones	43 (35.0%)
	Listening via PLD (earphones)	110 (89.4%)
	Music while travelling	78 (63.4%)
	Music while dancing	27 (22.0%)
	Music while studying	15 (12.2%)

Note: Activities are categorized by type. Values indicate the number and percentage of participants reporting each activity.

### PLD usage patterns

Among PLD users, earphones were the most used device (89.7%), followed by loudspeakers (39.7%) and headphones (34.9%). Earbud-style designs were preferred over traditional wired alternatives by many students. Approximately 29% of users reported using devices with Active Noise Cancellation (ANC) features. Fig. 1 illustrates the listening duration per day, the self-reported volume level, and the years of PLD usage.

**Fig. 1:**
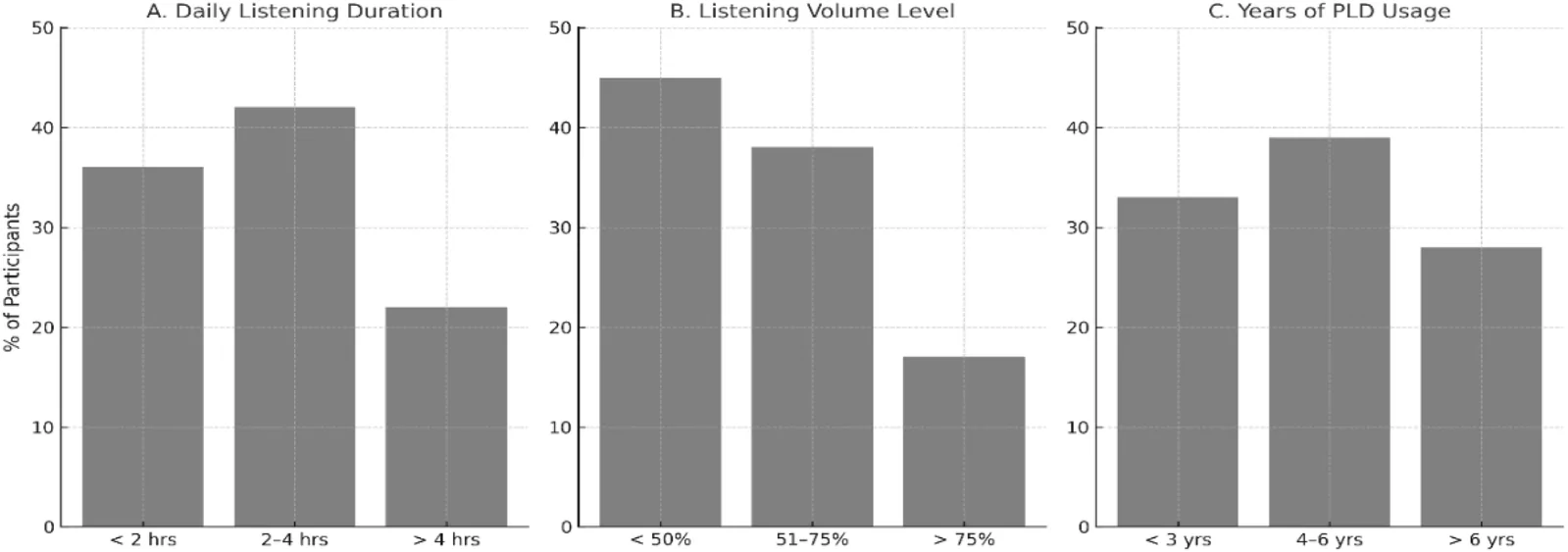
PLD usage patterns among participants by listening duration, volume level, and years of exposure (n = 123). (A) Daily listening duration; (B) Self-reported volume level; (C) Years of PLD use. Bars represent the percentage of participants in each category.

### Noise exposure levels

Using the ENHDC questionnaire, cumulative and perceived exposure levels were estimated. Activities such as fireworks, movies in theatres, and nightclubs exhibited the highest median sound levels (100 dBA), followed by concerts and playing in a band (90 dBA). Earphones and headphones contributed the highest cumulative exposure scores (81.2 and 75.4, respectively), reflecting longer and more frequent usage. Activities like television viewing, studying with music, and travelling with music, although lower in median Sound Pressure Level (SPL) (70–80 dBA), showed meaningful cumulative exposure due to duration and frequency (Table 2).

**Table 2:** Median estimated noise exposure levels (dBA), cumulative exposure scores, and years of exposure across selected activities (n = 123).

Activity	Median SPL (dBA)	IQR (dBA)	Median Cumulative Exposure	IQR	Median Years	IQR
Playing on stage/orchestra	90.00	10.00	71.60	14.00	5.00	8.00
Musical instruments	70.00	0.00	63.60	7.94	3.00	5.00
Loudspeakers	80.00	17.50	67.30	17.00	5.00	4.00
Headphones	80.00	10.00	75.40	15.40	3.00	3.00
Earphones	80.00	7.50	81.20	13.20	4.00	3.00
Watching television	70.00	10.00	77.20	14.30	15.00	5.00
Playing games	70.00	10.00	65.90	19.90	4.00	4.00
Watching movies/ theatre	100.00	10.00	84.60	10.80	10.00	8.50
Nightclubs/pubs	100.00	10.00	76.70	12.30	2.00	2.00
Concerts/festivals	90.00	10.00	76.10	11.80	10.00	10.00
Fireworks	100.00	20.00	78.60	20.00	15.00	5.00
Exercising to music	80.00	11.80	68.60	14.70	3.00	2.80
Music while travelling	70.00	10.00	72.40	12.10	5.00	7.80
Music while dancing	70.00	15.00	76.70	19.90	3.00	2.00
Music while studying	70.00	10.00	68.80	15.70	4.00	2.00

SPL, Sound Pressure Level; IQR, Interquartile Range.

Values are based on self-reported responses in the ENHDC questionnaire.

### Audiological test results

Most participants had normal hearing thresholds (≤ 20 dB HL), although greater variability was observed in the high and extended high-frequency ranges. The Shapiro–Wilk test showed that most measures were non-normally distributed (p < 0.05), except for the average of Noise Exposure scores (NE AVG) (*W* = 0.990, p = 0.670). The Pure Tone Average (PTA) thresholds were 10.00 dB HL (IQR = 5.00) in both ears, ranging from 1.67 to 28.33 dB HL in the right ear and −3.33 to 18.33 dB HL in the left. Elevated thresholds were noted in the High-Frequency Average (HFA: median = 8.33 dB HL, IQR = 6.67) and in the Extended High-Erequency Average (EHFA: right ear median = 5.63 dB HL, left ear median = 8.75 dB HL).

Speech Perception in Noise (SPIN) scores (Median = 1.13 dB SNR, IQR = 0.199) reflected the signal-to-noise ratio at 50% correct (SNR-50) threshold, while Temporal Fine Structure – Adaptive Frequency (TFS-AF) thresholds had a median of 997 Hz (IQR = 587). Amplitude Modulation Detection (AMD) thresholds showed a median of −15.10 dB (IQR = 4.53), consistent with normal modulation sensitivity. A summary of all variables is provided in Table 3.

**Table 3:** Summary of audiological thresholds, listening habits, and auditory processing outcomes among participants (n = 123).

Variable	Median (IQR)	Min	Max	Shapiro Wilk p
Age (years)	21.00 (3.50)	18.00	26.00	<0.001
PLD – Hours/day	2.00 (3.00)	0.25	18.00	<0.001
PLD – Volume Level (%)	70.00 (22.50)	30.00	100.00	0.003
PTA Right (dB HL)	10.00 (5.00)	1.67	28.33	0.001
PTA Left (dB HL)	10.00 (5.00)	−3.33	18.33	0.004
HFA Right (dB HL)	8.33 (6.67)	−5.00	38.33	<0.001
HFA Left (dB HL)	8.33 (6.67)	0.00	35.00	<0.001
EHFA Right (dB HL)	5.63 (11.90)	−9.38	53.60	<0.001
EHFA Left (dB HL)	8.75 (10.60)	−8.75	46.30	0.001
TFS AF Score (Hz)	997.00 (587.00)	108.00	2023.00	<0.001
SPIN Score (dB SNR)	1.13 (0.199)	0.54	1.45	<0.001
AMD Score (dB)	−15.10 (4.53)	−19.90	−5.78	<0.001
NE AVG Total*	74.90 ± 4.96	64.10	87.80	0.670

All values presented as Median (IQR) unless indicated. Noise Exposure Average (NE AVG) was normally distributed; mean (SD) is reported. PTA, Pure Tone Average; HFA, High Frequency Average; EHFA, Extended High Frequency Average; TFS-AF, Temporal Fine Structure-Adaptive Frequency; SPIN, Speech Perception in Noise; AMD, Amplitude Modulation Detection; PLD, Personal Listening Device; IQR, Interquartile Range.

### Correlation between noise exposure and auditory processing

To account for differing scales, *z*-scores were computed for SPIN, TFS-AF, AMD, and overall noise exposure prior to correlation analysis. Spearman’s rho values revealed no significant associations between cumulative noise exposure and any auditory processing measure (TFS-AF: ρ = −0.07, p = 0.44; SPIN: ρ = −0.12, p = 0.18; AMD: ρ = −0.11, p = 0.24). However, a moderate positive correlation was observed between SPIN and AMD scores (ρ = 0.23, p = 0.01), suggesting that individuals with better speech-in-noise perception also showed stronger modulation detection ability (Table 4).

**Table 4:** Spearman’s correlations between auditory processing measures and cumulative noise exposure (*z*-scores, n = 123).

Correlation Matrix					
		Noise Exposure	TFS-AF	SPIN	AMD
**Noise Exposure**	Spearman's rho	‒			
	*df*	‒			
	p-value	‒			
**TFS-AF**	Spearman's rho	−0.07	‒		
	*df*	122	‒		
	p-value	0.44	‒		
**SPIN**	Spearman's rho	−0.12	0.08	‒	
	*df*	122	121	‒	
	p-value	0.18	0.33	‒	
**AMD**	Spearman's rho	−0.11	0.06	**0.23**	‒
	*df*	122	121	121	‒
	p-value	0.23	0.53	**0.01**	‒

Values represent Spearman’s ρ. p < 0.05 marked in bold. *Z*-scores used for normalization across metrics.

### Group-wise comparisons by noise exposure level

To assess the impact of noise exposure level, participants were grouped into quartiles based on cumulative exposure scores. The highest (Q4) and lowest (Q1) quartiles (n = 32 each) were compared using Mann-Whitney *U*-tests, and effect sizes were reported as *r* (Table 5).

**Table 5:** Group comparisons between high (Q4) and low (Q1) noise exposure quartiles using Mann-Whitney *U*-test (n = 64).

Variable	*U*	p	Mean Difference	Effect Size (r₍rb₎)
Noise Exposure	23.00	<0.001	13.09	0.96
SPIN	354.50	0.035^a^	0.06	0.31
TFS-AF	486.50	0.737	20.66	0.05
AMD	405.00	0.153	1.15	0.21
PTA – Right	341.00	0.021^a^	3.33	0.33
PTA – Left	346.00	0.024^a^	1.67	0.32
HFA – Right	414.00	0.187	1.67	0.19
HFA – Left	416.50	0.200	1.67	0.19
EHFA – Right	480.50	0.677	1.25	0.06
EHFA – Left	485.50	0.727	0.63	0.05
PLD Volume (%)	410.50	0.169	5.85	0.20
PLD Hours/Day	353.00	0.030^a^	1.00	0.31

PLD, Personal Listening Device; PTA, Pure Tone Average; HFA, High Frequency Average; EHFA, Extended High Frequency Average. p < 0.05 marked with^a^. Effect sizes are reported as rank biserial correlation.

As expected, a statistically significant difference was observed in cumulative noise exposure levels between the two groups (*U* = 23.00, p < 0.001, *r* = 0.96), confirming the intended separation of exposure categories. The high-exposure group also demonstrated poorer SPIN scores compared to the low-exposure group (*U* = 354.50, p = 0.035, *r* = 0.31). Similarly, pure-tone thresholds were significantly elevated in the high exposure group for both ears (PTA-R: *U* = 341.00, p = 0.021, *r* = 0.33; PTA-L: *U* = 346.00, p = 0.024, *r* = 0.32).

No significant differences were observed for TFS-AF (*U* = 486.50, p = 0.737), AMD (*U* = 405.00, p = 0.153), or EHFA (EHFA-R: p = 0.677; EHFA-L: p = 0.727).

Additionally, daily personal PLD usage differed significantly, with the high-exposure group reporting higher listening durations (*U* = 353.00, p = 0.030, *r* = 0.31); however, no significant group difference was found in reported PLD volume settings (*U* = 410.50, p = 0.169). Group-wise means and confidence intervals are visualized in Fig. 2.

**Fig. 2:**
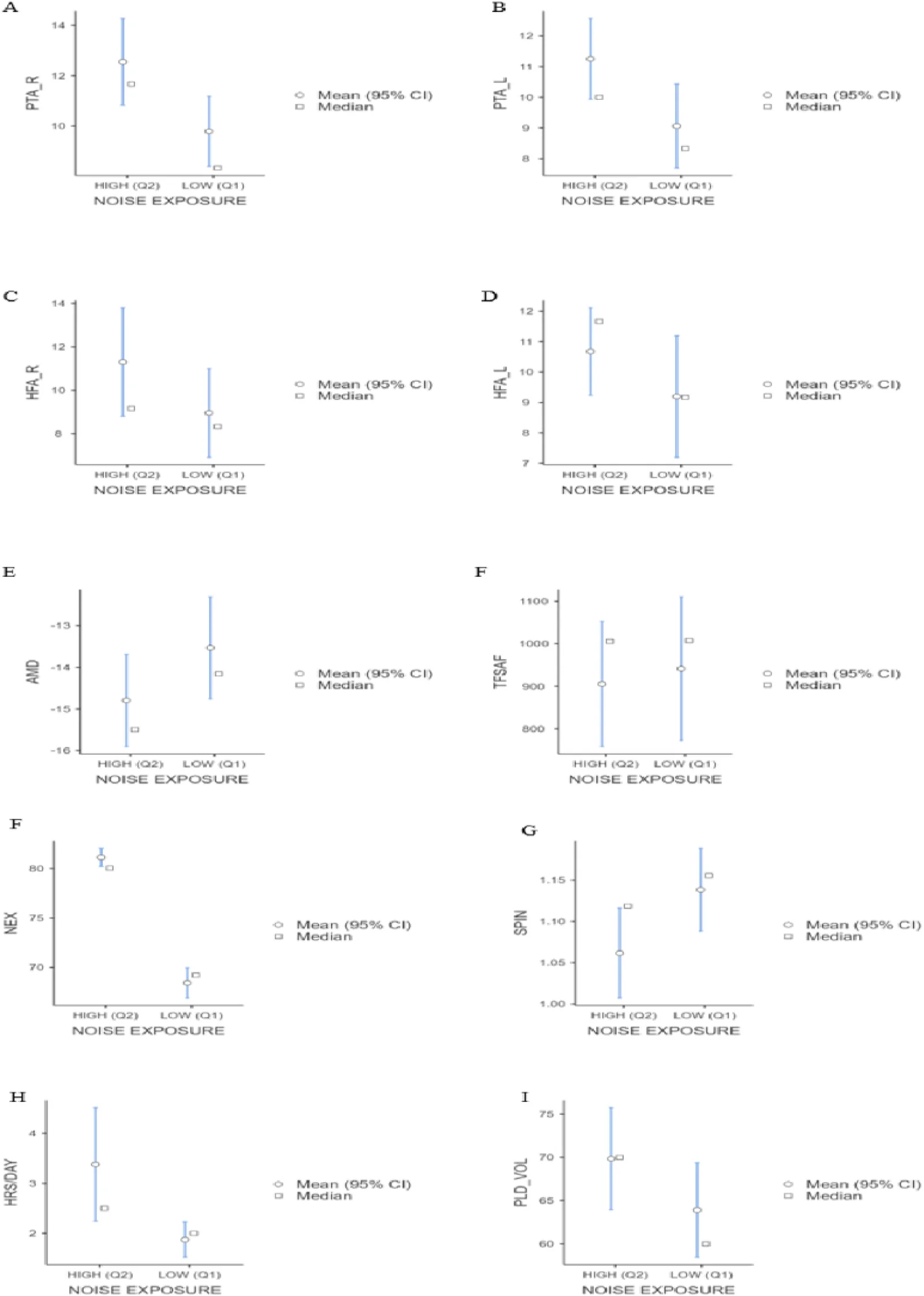
Group-wise comparisons between low (Q1) and high (Q4) recreational noise exposure participants across various auditory and behavioral measures. (A‒B) Pure-tone average thresholds (right and left ears); (C‒D) High-frequency average thresholds (right and left ears); (E) Amplitude Modulation Detection (AMD) thresholds; (F) Temporal Fine Structure – Adaptive Frequency (TFS-AF); (G) Cumulative recreational Noise Exposure Score (NEX); (H) Speech Perception in Noise (SPIN) performance; (I) Daily Personal Listening Device (PLD) usage in hours; (J) Self-reported PLD listening volume levels. Circles represent mean values; squares represent medians. Error bars denote 95% Confidence Intervals. (PTA, Pure-Tone Average; HFA, High-Frequency Average; EHFA, Extended High-Frequency Audiometry; IQR, Interquartile Range).

To validate the group differences across all quartiles, a Kruskal-Wallis H test was also conducted on the full sample (n = 123). Significant differences were observed for cumulative noise exposure (χ^2^ = 43.11, p < 0.001), SPIN (χ^2^ = 4.48, p = 0.034), PTA thresholds (PTA-R: χ^2^ = 5.36, p = 0.021; PTA-L: χ^2^ = 5.10, p = 0.024), and PLD hours/day (χ^2^ = 4.76, p = 0.029).

## Discussion

The present study investigated the impact of recreational noise exposure on auditory processing in college students with clinically normal hearing thresholds. Findings revealed that cumulative recreational noise exposure is associated with subtle yet measurable deficits in auditory function, particularly in speech-in-noise perception and marginal elevations in pure tone thresholds. These results support the hypothesis that standard audiometric evaluations may not effectively detect early noise-induced auditory changes in young adults, reinforcing concerns about “hidden hearing loss” in this group.^21^^,^^22^

The recreational noise exposure patterns identified in this study indicate that college students face significant auditory risks from leisure activities. Personal Listening Devices (PLD) usage was the prevailing source of exposure, reported by 90% of the subjects. Duration of PLD usage was significantly linked to higher cumulative noise exposure, aligning with prior studies that highlight duration as a primary factor in noise dose and auditory stress.^6^^,^^9^ Although group differences in volume levels were not statistically significant, the higher proportion of moderate to high volume usage among the high exposure group supports concerns raised by^10^ and^13^ regarding volume preference as a risk factor for early cochlear damage. Twenty-two per cent of participants reported listening for over 4-hs daily, and 17% exceeded 75% of maximum volume, suggesting that young adults engage in potentially hazardous listening behaviours, consistent with findings from previous studies.^6^ The risk of exceeding recommended exposure levels with typical PLD use has been documented in recent cohort studies.^14^

Participants in the high-exposure group demonstrated elevated Pure Tone Average (PTA) thresholds, although still within the clinically normal range. Literature also reported early-stage cochlear stress in noise-exposed young adults.^30^ Similarly, while extended high-frequency thresholds showed a non-significant increase in the high exposure group, this may reflect subtle outer hair cell dysfunction not yet detectable by standard audiometry, as discussed by Mehrparvar et al. (2011).^31^ Recent normative datasets also highlight the sensitivity of extended high-frequency thresholds to early auditory changes in young adults.^32^^,^^33^ It has also been shown to correlate with speech-in-noise difficulties in young adults with clinically normal hearing.^34^ Taken together, these findings suggest that extended high-frequency audiometry may serve as an early indicator of basal cochlear vulnerability that is not captured by conventional pure-tone measures.

Additionally, Speech Perception in Noise (SPIN) performance was significantly poorer in the high exposure group, suggesting functional difficulties with suprathreshold speech processing. These findings align with previous studies^21^^,^^24^ that demonstrated noise-induced deficits in temporal coding or neural synchrony may manifest as difficulty understanding speech in complex environments. This deficit occurred despite normal audiometric thresholds, suggesting that noise-induced damage may preferentially affect neural processing mechanisms underlying complex auditory tasks.

The absence of significant differences in Temporal Fine Structure – Adaptive frequency (TFS-AF) and Amplitude Modulation Detection (AMD) scores suggests recreational noise exposure at observed levels may not substantially impact temporal fine structure processing, or these tests may be less sensitive to recreational exposure-specific damage.^22^ The preserved TFS processing alongside impaired speech-in-noise perception also suggests that different aspects of auditory processing may be differentially affected by noise exposure, with speech perception in complex acoustic environments being more vulnerable to recreational noise-induced changes. Recent findings suggest that temporal processing abilities substantially contribute to speech-in-noise perception, even in listeners with normal audiograms.^35^ Further research is necessary to delineate the underlying mechanisms and assess the sensitivity of current measures in detecting early noise-induced auditory changes.

The overall pattern of results supports the growing body of literature suggesting that recreational noise exposure, even in young adults with normal audiograms, can induce early, subclinical changes in auditory processing. These effects may particularly impact daily listening in noisy or acoustically demanding environments, as reflected by subtle group differences in SPIN and AMD performance. The study thus reinforces the importance of using ecologically valid behavioral tests to detect functional auditory deficits that precede audiometric threshold shifts.

### Clinical implication

These findings have several clinical implications. Subtle shifts in Extended High-Frequency Audiometry (EHFA) thresholds and reduced Speech-In-Noise (SPIN) performance, even in the presence of normal conventional audiometric thresholds, may indicate early cochlear or neural stress associated with recreational noise exposure. Incorporating EHFA and ecologically valid SPIN measures into routine assessments for high-risk groups, such as college students with heavy personal listening device use, may improve the early detection of subclinical auditory changes that precede overt threshold shifts. The present findings also highlight the need for education and counselling regarding safe listening practices and for increasing awareness of Recreational Noise-Induced Hearing Loss (RNIHL) and Hidden Hearing Loss (HHL), particularly in youth-centred environments. Also, where early signs of auditory stress are identified, individualized hearing conservation strategies may be considered to prevent progression to permanent auditory damage.

### Limitations and future directions

The study has a few limitations. The sample was drawn from a large and diverse student population; however, the overall size may not fully capture the variability in recreational listening behaviors, lifestyle factors, or auditory profiles present in the broader young adult population. This may limit the generalizability of the findings. Additionally, the use of a self-reported noise exposure questionnaire introduces potential recall bias and subjective variability, which may impact the precision of cumulative exposure estimates. Additionally, the current study ruled out recent noise exposure solely through case history. There was no standard auditory rest period in the protocol which may not fully rule out short-term auditory effects. Future research should incorporate objective monitoring tools such as real-time noise dosimetry or app-based tracking to enhance exposure accuracy.

## Conclusion

The present study highlights the subtle yet significant impact of recreational noise exposure on young adults’ auditory function, even before clinically apparent hearing loss emerges. The findings indicate the significance of including suprathreshold auditory measures, such as SPIN and EHFA, in routine screening protocols for at risk populations. The findings highlight the need for public health initiatives to educate young adults about safe listening practices and the long-term effects of noise exposure. They emphasize incorporating preventive audiology strategies into early identification efforts to protect hearing health.

## ORCID ID

Visishta Thavarayil: 0009-0007-3018-8605

## Authors’ contributions

Visishta Thavarayil: Writing-original draft; visualization; validation; methodology; investigation; formal analysis; data curation; conceptualization.

Leelavathi Thamizhmani: Writing-review & editing; validation; conceptualization.

Kanaka Ganapathy: Supervision; resources; writing-review & editing; methodology; and conceptualization.

Hari Prakash Palaniswamy: Writing-review & editing; methodology; formal analysis.

## Informed consent

Written informed consent was obtained from all participants prior to inclusion in the study.

## Ethical approval

The study adhered to the ethical guidelines outlined by the Institutional Ethics Committee (IEC) (IEC2:345/2024) of Kasturba Medical College (KMC) and Kasturba Hospital (KHC), Manipal, India. These guidelines align with the National Ethical Guidelines for Biomedical Research on Human Participants issued by the Indian Council of Medical Research (ICMR, 2017), which adhere to the principles of the Declaration of Helsinki. The study was registered with the Clinical Trials Registry of India (CTRI/2024/08/072299). Written informed consent was obtained from all participants prior to their inclusion in the study.

## Funding

This research did not receive any specific grant from funding agencies in the public, commercial, or not-for-profit sectors.

## Data availability

The datasets generated and/or analysed during the current study are available from the corresponding author on reasonable request.

## Declaration of competing interest

The authors declare no conflicts of interest.
